# Biomimetic and Live Medusae Reveal the Mechanistic Advantages of a Flexible Bell Margin

**DOI:** 10.1371/journal.pone.0048909

**Published:** 2012-11-07

**Authors:** Sean P. Colin, John H. Costello, John O. Dabiri, Alex Villanueva, John B. Blottman, Brad J. Gemmell, Shashank Priya

**Affiliations:** 1 Marine Biology/Environmental Sciences, Roger Williams University, Bristol, Rhode Island, United States of America; 2 Whitman Center, Marine Biological Laboratory, Woods Hole, Massachusetts, United States of America; 3 Biology Department, Providence College, Providence, Rhode Island, United States of America; 4 Graduate Aeronautical Laboratories and Bioengineering, California Institute of Technology, Pasadena, California, United States of America; 5 Center for Energy Harvesting Materials and Systems (CEHMS), Virginia Tech, Blacksburg, Virginia, United States of America; 6 Naval Undersea Warfare Center, Division Newport, Newport, Rhode Island, United States of America; University of Cambridge, United Kingdom

## Abstract

Flexible bell margins are characteristic components of rowing medusan morphologies and are expected to contribute towards their high propulsive efficiency. However, the mechanistic basis of thrust augmentation by flexible propulsors remained unresolved, so the impact of bell margin flexibility on medusan swimming has also remained unresolved. We used biomimetic robotic jellyfish vehicles to elucidate that propulsive thrust enhancement by flexible medusan bell margins relies upon fluid dynamic interactions between entrained flows at the inflexion point of the exumbrella and flows expelled from under the bell. Coalescence of flows from these two regions resulted in enhanced fluid circulation and, therefore, thrust augmentation for flexible margins of both medusan vehicles and living medusae. Using particle image velocimetry (PIV) data we estimated pressure fields to demonstrate a mechanistic basis of enhanced flows associated with the flexible bell margin. Performance of vehicles with flexible margins was further enhanced by vortex interactions that occur during bell expansion. Hydrodynamic and performance similarities between robotic vehicles and live animals demonstrated that the propulsive advantages of flexible margins found in nature can be emulated by human-engineered propulsors. Although medusae are simple animal models for description of this process, these results may contribute towards understanding the performance of flexible margins among other animal lineages.

## Introduction

Medusae are thought to be the first metazoans to evolve muscular swimming. The simplicity yet functionality of the biomechanics of their swimming make them an ideal organismal model for studying fundamental mechanisms of animal propulsion. Many medusan species swim using rowing propulsion [1], [2], [3]. To do so, they rhythmically contract epitheliomuscular cells which line the under, or subumbrellar, surface of their bells. However, these circular muscles do not extend to the tips of the bell (R. Satterlie, pers.comm.) and so as the bell contracts and expands the highly flexible bell margin undergoes substantial flapping deformations during these cyclic movements. These motions generate large starting and stopping vortices during the contraction and expansion phases of the cycle, respectively [2]. The generation and interaction of these vortices provides thrust for swimming and transports large volumes of fluid through capture structures for feeding [4], [5], [6]. Additionally, the interaction of the starting and stopping vortices reduces the energy transferred to the surrounding fluid thereby enhancing the efficiency of rowing propulsion [2], [4], [7], [8]. The influence of bell margin flexibility on vortex formation and thrust production at the bell margin has not been evaluated but has emerged as a critical characteristic of rowing propulsion and for the design of robotic jellyfish vehicles.

Existing literature provides a conflicting picture of the potential interactions between propulsor flexibility and swimming thrust production. Empirical [9], [10], modeling [11], [12], [13], and computational studies [14], [15], [16] have indicated that, under some circumstances, flexible margins may augment thrust production during swimming and flying. However, the range of conditions under which propulsor flexibility is advantageous vary [17] and, although a range of vehicles have recently incorporated flexible propulsor margins in an effort to maximize propulsive proficiency [18], [19], guidelines for the advantageous application of flexibility to propulsors have remained elusive. Flexibility is an important trait of many natural propulsors [17], [20], but no general principles describing the function of nature's flexible propulsors have emerged that could serve to guide engineering applications.

An important reason for the absence of design criteria guiding application of flexible propulsor tips or margins is that, despite an extensive body of work on flexible propulsors, the mechanistic basis for thrust augmentation by flexible propulsor margins remains unclear [21]. Documentation of the underlying process is hindered by the inherently unsteady nature of these effects [22], [23] and the frequently complex fluid interactions occurring within the wakes of multiple, interacting propulsive structures [24], [25]. Consequently, the rich literature on this topic has provided a wide range of information for specific conditions, but limited general predictive capacity for design of flexible propulsors. Our goal in this work was to develop a mechanistic description of the physical basis of thrust augmentation by the flexible bell margins of medusae, and by extension, vehicles inspired by medusan propulsion.

**Figure 1:**
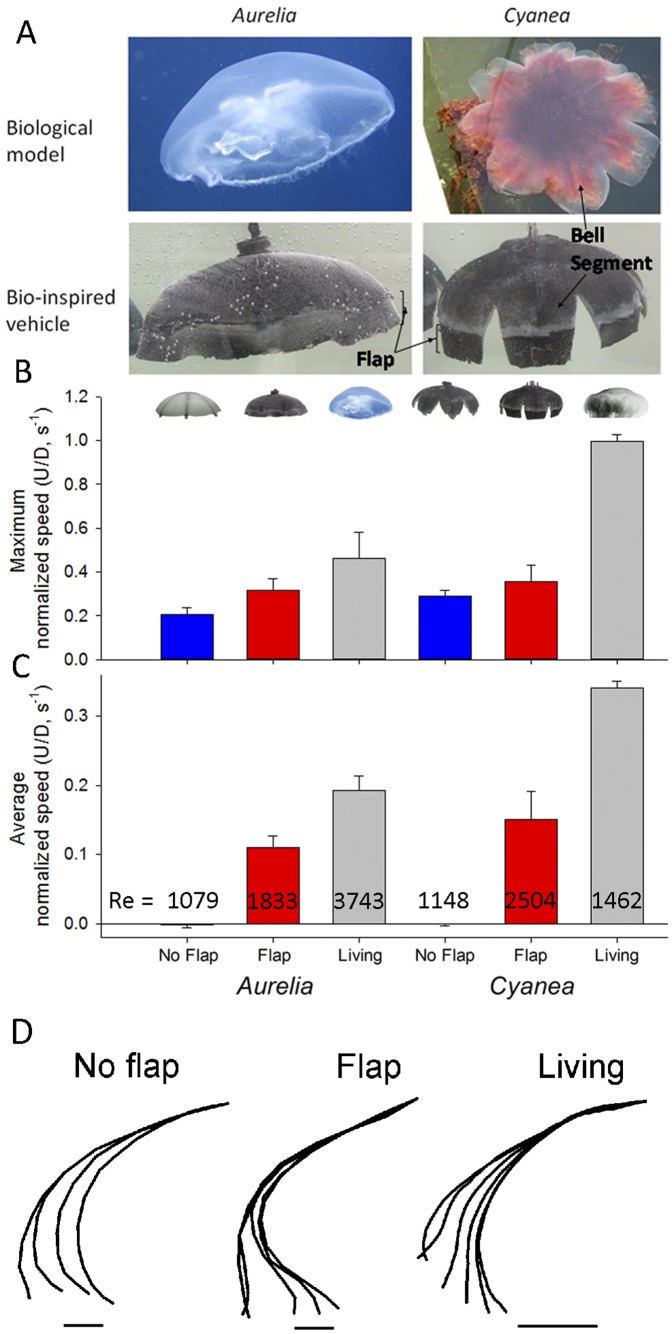
Performance among robots and live medusa. A. Structural comparison of biological models (top panels) with bio-inspired vehicles (bottom) used for experimental comparisons (both are 16.4 cm diameter). Only flap versions of the robotic vehicles are shown. B. Comparison of the maximum normalized swimming speeds of jellyfish vehicles with representative values for their natural counterparts, *Aurelia aurita* (14.7 cm diameter) and *Cyanea capillata* (10 cm diameter). C. Comparison of the normalized swimming speed averaged over the swimming cycle. The Reynolds numbers (Re) shown are averages over 3 swimming cycles. We examined a flap and a no-flap version of both the *A. aurita* and *C. capillata* vehicle models (icons illustrate each version). Bars are the mean values over 3 consecutive swimming cycles (± st.dev.). D. Bell kinematics at of no-flap and flap vehicles and live *Aurelia* during bell contraction. Scale represents 1 cm.

A recently developed robotic jellyfish vehicle (Fig. 1A) has provided us with another tool, in addition to empirical, modeling and computational approaches, to understand how propulsor kinematics determine swimming performance [26], [27]. The structural and functional simplicity of medusan architecture provides valuable advantages for comparative use of robotic models with live animals. The medusan bell is a simple, axisymmetric form with a flexible margin and without other secondary structures that generate complex interacting vortices. The robotic jellyfish is comprised of a flexible silicone bell that is actuated by bio-inspired shape memory alloy composite actuators (BISMAC) that contract when an electrical current is applied and return to their original position when the current is turned off [26], [28]. With these simple components, the kinematics of the robotic jellyfish are able to replicate the kinematics of natural medusae [26], [29], [30]. An important advantage of a robotic vehicle is that, although the comparative approach using live species has yielded important insights (e.g.; [7]), more controlled experiments are possible by manipulating the robotic design and actuation.

Our approach to determining the mechanistic basis of thrust augmentation by flexible propulsor margins was to compare propulsion by robotic jellyfish vehicles with and without flexible bell margins to each other and to live medusae (Fig. 1A). With the robotic approach, we can attribute differences in swimming performance between vehicle versions solely due to the presence or absence of a passive, flexible flap along the bell margin of the robotic vehicles. Similar manipulation via addition or removal of components to live animals would be difficult without introducing elevated physiological stress as an unwanted variable. Importantly, our use of vehicular analogues to medusae allowed alteration of vehicle components to test structure-function hypotheses while maintaining constant power input to all test vehicles.

## Materials and Methods

Robotic jellyfish vehicle fabrication and function was designed to emulate the flexible motions characteristic of jellyfish bells during swimming. Two vehicles of similar construction were used during experimentation. One vehicle had a continuous bell emulating *Aurelia aurita*. The other was modified to represent the *Cyanea capillata* morphology by excising portions of the bell to form gaps along the bell margin (Fig. 1A). Experiments have shown that the robotic bell mimics the cyclic deformation and hysteretic curvature changes of live medusae (Fig. 1D) [26], [29], [30]. The details of jellyfish vehicle construction and comparisons of the kinematics of the bell motions of the vehicle with natural medusae are described by Villanueva et al. (2010a; 2010b; 2011). Briefly, the mesoglea was fabricated from soft silicone material with tensile elastic modulus of 56 KPa [30](live jellyfish have elastic modulus on the order of 0.9 MPA [31]) and physical dimensions (bell diameter = 164 mm) within the range of their natural counterpart. Detailed description of physical properties of the materials used for construction of all the components can be found elsewhere [32]. The propulsive muscles consisted of BISMAC actuators arranged radially around the bell at angles of 45° from each other. Extensive computational and structural modeling of robotic jellyfish has been conducted to optimize the transient deformation behavior at BISMAC and segmented locations, tune the effectiveness of the joint design, and understand the role of material variables in robotic jellyfish [32]. The passive region along the bell margin, referred to as the flap, was constructed from the same silicon material as the rest of the bell. For the experiments comparing the flap versus no-flap, the flap dimensions replicated the dimensions of the passive region observed in live medusae based upon digitized bell kinematics during swimming. For the experiments comparing the effect of flap length on performance, incrementally longer flaps were attached to the robotic vehicle ranging from 7.2 to 25 mm. All vehicle comparisons were performed using equal power input to the vehicles (ANOVA comparison of recorded power consumption (current×voltage) during pulse cycles; p>0.2). Vehicles were powered with a square wave input of 0.7 Hz frequency, duty cycle of 20%, voltage amplitude of 90 V and offset of 45 V. Consequently, comparison among flap versus no-flap and variable flap lengths involved the same vehicles with identical power inputs but different bell margin configurations.

**Figure 2:**
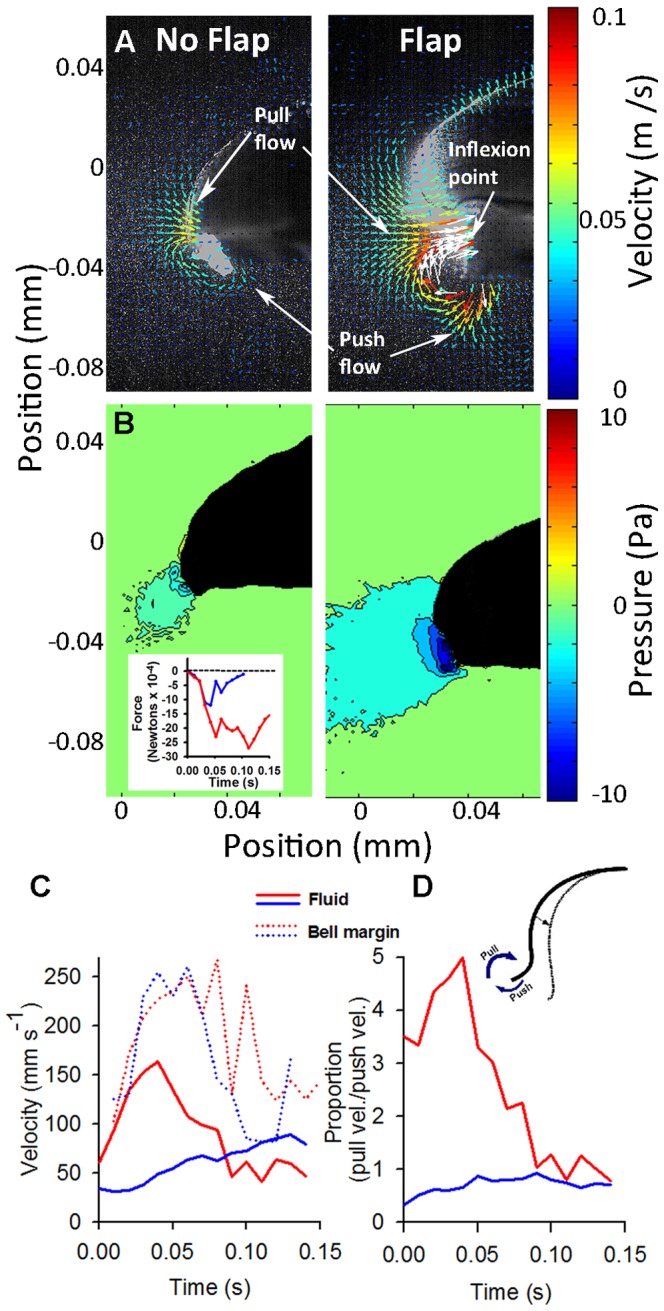
Fluid interactions at the bell margin. (A) Velocity vector field (from DPIV record) and (B) pressure field of flows around flap and no-flap versions of the *Aurelia* vehicle. Inset: Area integrated pressure of the pressure field along the bell over time. In the flap version, the greatest fluid velocities and lowest pressure values occurred at the inflexion point of the margin, where the flexible flap joined the more rigid bell. (C) Maximum fluid velocities in the wake were greater for the flap version (red) of the *Aurelia* vehicle than the no-flap (blue) version although the velocities of the propulsors (bell margins) did not differ. (D) Ratio of the maximum velocities of the fluid entrained (pull) versus expelled (push) by the bell during bell contraction for the flap (red) and no-flap (blue) versions. Velocity and pressure fields of the *Aurelia* vehicle were representative of the fields for the *Cyanea* vehicle.

All video recordings of the robotic vehicles were conducted in a 130 gallon glass aquarium containing fresh water at room temperature. We used two-dimensional digital particle image velocimetry (DPIV) to quantify vehicle kinematics as well as fluid motions [7] surrounding medusae vehicles and live medusae. As medusae pulse they generate distinct starting and stopping vortex rings during the power and recovery strokes of the swim cycle, respectively [2]. The radial symmetry of medusae enables us to assume the wake structures are axisymmetric and enable us to use two-dimensional analyses for comparisons among vehicles and medusae. Three-dimensional DPIV has confirmed that the flows around straight swimming medusae are axisymmetric (unpublished data). To quantify the flow, vehicles were placed in water seeded with 10 µm hollow glass beads, then illuminated by a laser sheet (680 nm wavelength) while recording (500–1000 frames-per-second) with a high-speed digital video camera (Photron Fastcam 1024 PCI) during swimming. The velocities of particles illuminated in the 2D laser sheet were determined by analyzing sequential images using a cross-correlation algorithm (DaVis software, LaVision Inc., Ypsilanti, USA). Image pairs were analyzed with shifting overlapping interrogation windows of decreasing size (64×64 pixels then 32×32 pixels). We measured the circulation (Γ) of the starting vortex ring which was defined as the vorticity (ω) integrated over the area of the vortex ring,
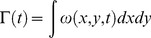
(1)where *x* and *y* represent spatial coordinates over a time interval *t*. We also extracted specific velocity values from the regions around the medusae using the DaVis software. For maximum velocities in the wake, the measured area encompassed the entire bell margin region (including both the ‘push’ and ‘pull’ regions; see inset in Fig. 2). For calculating the ratio of the velocities in the push and pull region we extracted maximum velocities from regions that completely encompassed the flow in the ‘push’ and ‘pull’ regions (see inset in Fig. 2).

Swimming kinematics were quantified by using ImageJ software (NIH) to track the *x* and *y* coordinates of the apex of the vehicle bell and the tips of the bell margin over time. Swimming speed was calculated from the change in the position of the apex over time as
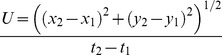
(2)The bell fineness ratio was defined as *h*/*d* where *h* represents bell height and *d* represents bell diameter.

Pressure field data was inferred from the measured velocity fields by numerically integrating the inviscid Navier-Stokes equation, or Euler equation:
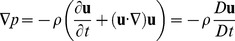
(3)where ρ is the fluid density and **u** is the Eulerian velocity field.

The material acceleration term 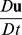, which quantifies the acceleration of individual fluid particles in the flow, was calculated from the measured DPIV velocity field **u**(*x*, *y*). The pressure term was then determined to within a constant of integration by integrating equation (3) spatially.

To reduce errors in the numerical integration of the measured velocity data, the procedure of Liu and Katz [33] was employed. Their algorithm integrates equation (3) along multiple paths from the boundary of the domain to each point in the flow and averages the calculated pressures along each path. Because the pressure at each point in space should be independent of integration path, each path integration is effectively an independent estimate of the local pressure at the points along a path. The average of these estimates therefore reduces random errors that accumulate along individual integration paths.

The input to the version of the code used presently (kindly provided by X. Liu) took as input a time series of DPIV data on a 128×128 grid. Preprocessing of the DPIV data was completed in MATLAB to compute the material acceleration 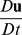, which is also a required input to the pressure calculation code. Whereas Liu and Katz (2006) utilized an iterative method to estimate the material acceleration from four flow images collected alternately by two cameras, here we used an approximation based on two successive DPIV velocity fields collected from a single camera. To determine the material acceleration, we computed the difference in the velocity of fluid particles initially located at the DPIV data grid points 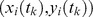 at time *t_k_* and subsequently advected to new positions 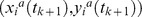 at time *t_k_*_+1_. The initial velocity of each fluid particle is given by the measured DPIV velocity field at time *t_k_* (i.e. *i* = 1, 2,…128 presently; *k* is the DPIV time step number). The velocity of each fluid particle at time *t_k_*_+1_ depends on the measured DPIV velocity field at time *t_k_*_+1_ and on the position of the fluid particle at the same instant in time. To determine the position of each fluid particle in the flow at time *t_k_*_+1_, we defined an advection velocity field in which the fluid particles are carried. The advection velocity field was taken to be the average of two successive DPIV velocity fields:
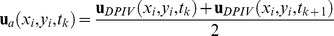
(4)where the advection velocity field **u** *_a_* was assigned to the first time instant *t_k_* by the present convention. The positions of the advected data grid points at time *t_k_*_+1_ are therefore estimated as
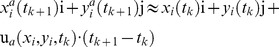
(5)where **i** and **j** are unit vectors in the *x*- and *y*-directions, respectively. The material acceleration was then estimated as
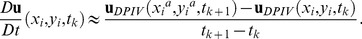
(6)This choice of approximation was deemed suitable due to the relatively slow temporal evolution of the flow.

As a check of the dependence of the results on viscous stresses, which are neglected in equation (3), the pressure was also calculated using the full Navier-Stokes equations with the viscous term estimated from the two-dimensional DPIV data. The result were qualitatively consistent, albeit with greater noise in the pressure calculation due to errors introduced by taking two spatial derivatives of the DPIV data in order to compute the Laplacian term 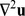.

The output of the code was a time series of pressure fields with scalar pressure computed at each of the 128×128 nodes of the corresponding DPIV fields.

Live medusae originated from cultured collections of the New England Aquarium, Boston, MA. Video and DPIV methods were the same for both live animals and medusan vehicles except that the live medusae were video recorded in filtered seawater.

Due to the intensive effort involved in manual manufacturing of robotic vehicles we were limited in terms of number of the *Aurelia* and *Cyanea* vehicles. While we did not treat the two vehicles as replicates, because of their different bell configurations, the flap and no-flap versions of each do provide repeatable validation of the effects of a flexible propulsor on the fluid interactions and swimming performance of the vehicles. Means and variability estimates on figures with error bars were calculated by averaging over three consecutive swimming cycles.

## Results

**Figure 3:**
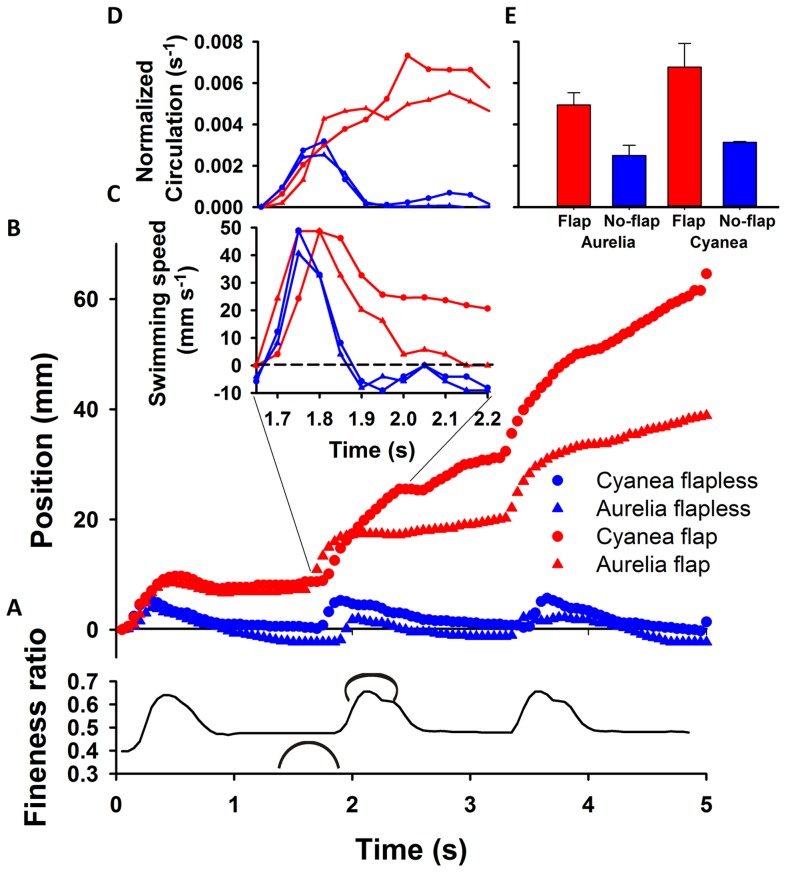
Vehicle performance and wake characteristics. (A) Swimming bell kinematics measured as changes in bell fineness (bell height/diameter) during contraction and relaxation (indicated by small medusa-shaped icons). (B) Corresponding propulsive performance of flap and no-flap vehicles supplied with identical input power. Although all vehicles accelerated forward during bell contraction, only vehicles with flexible margins – flaps – succeeded in making any net progress during bell relaxation. (C) Swimming speed during the second swimming cycle showing that the no-flap vehicles moved backwards (negative velocities) during bell expansion. (D) Circulation values of the starting vortex. Circulation values were normalized by bell area to account for differences in propulsor surface areas among vehicle versions. The increase in circulation occurs during bell contraction as the starting vortex grows. Circulation of the flap versions peaked at higher levels as a result of generating larger starting vortices.

Biomimetic robotic jellyfish vehicles closely replicated the morphology (Fig. 1A and 1D) and approached the swimming proficiency (Fig. 1B and 1C) of the cosmopolitan scyphomedusae, *Aurelia aurita* and *Cyanea capillata*. The proficiency of swimming was quantified as the diameter-normalized swimming speed to account for differences in size between the live and robotic jellyfish, *U*/*D* where *U* is medusa velocity and *D* is relaxed bell diameter [7]. The swimming proficiencies of the flap vehicles were about one-third to one-half the proficiency of the live medusae. These slower speeds are likely the result of small differences in bell kinematics, such as the vehicle bells not contracting as completely at the live medusae [26], and drag differences along the exumbrellar surfaces. Addition of a passive, flexible flap to the bell margin of medusan vehicles dramatically increased vehicle swimming proficiency and was in fact necessary for the swimming robotic medusae to achieve net forward motion over time (Fig. 1C and Fig. 3B). Interestingly, the maximum speeds, achieved during bell contraction (Fig. 3), did not differ as dramatically as the swimming speed averaged over the entire swimming cycle (Fig. 1B and 1C). A closer examination of the swimming speeds over time among the vehicles reveals that this is because, while all the vehicles accelerated during bell contraction, the no-flap vehicles decelerated dramatically during bell expansion and even moved in reverse (Fig. 3). In contrast, vehicles with marginal flaps sustained higher forward velocities throughout the swim cycle and continually moved forward (Fig. 3). The relative thrust produced by each version of the vehicles can be approximated and compared by looking at the circulation of the starting vortices generated during contraction [34], [35]. Each of the circulation time series illustrate the intensification of the starting vortex as it is being fed fluid expelled from inside the bell and entrained from outside the bell as the bell contracts. However, the starting vortices of the flap versions peaked at greater total circulation than the no-flap versions (Fig. 3D). The greater circulation, and therefore thrust, of the flap versions was not due to surface area differences for the vehicles due to the added flaps, as demonstrated by circulation rates normalized by propulsor surface area (Fig. 3D). In fact, the surface area of the *Cyanea* flap version was less than that of both *Aurelia* versions (flap and no-flap).

**Figure 4:**
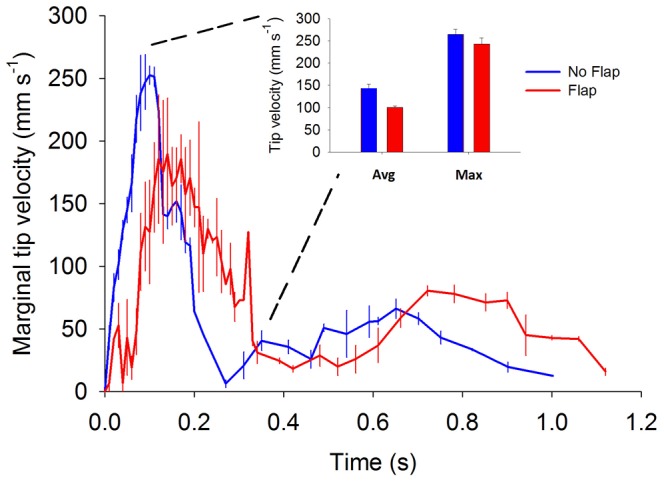
Comparison of margin tip velocities for flap and no-flap versions of the *Aurelia* vehicle. Data represent average (error bars - ±1 standard deviation of mean value) velocities taken during three consecutive pulsation cycles for each vehicle type. Insert depicts the average and maximum tip velocities for either vehicle type. Note that the vehicle possessing a flexible marginal flap did not have either higher maximum or average tip velocities than the vehicle without marginal flaps. The frequency of sampling was increased during the portion of the pulsation cycle characterized by maximum marginal tip velocities.

A closer look at the flow adjacent to the bell margin of the *Aurelia* robot during bell contraction demonstrates how the flexible margin contributed to the differences we observed in wake circulation and swimming performance. During bell contraction there were two sources of fluid momentum that contributed to the starting vortex circulation, the flow that was expelled, or pushed, from the subumbrellar volume and the flow that was entrained, or pulled, from outside the bell by the contracting bell margin (Fig. 2A). The maximum velocity of the fluid during contraction was much greater for the vehicle with the flap than for the no-flap version (Fig. 2C) and was located at and oriented toward the inflexion point of the flexible margin. Interestingly, the velocities of the tips of the bell margin of the flap and no-flap versions of the robot were not different (Fig. 4). Consequently, we cannot attribute the different wake velocities, and overall circulation, to differences in the speed of the tip of the bell margin during contraction. Another important difference observed in the flow adjacent to the bell margin was that a large majority of the flow contributing to the starting vortex of the flap versions came from the wake that was entrained, or pulled, from outside the bell while for the no-flap version there was roughly an equal contribution from the pulled and pushed flow (Fig. 2D).

Pressure calculations derived from the velocity vector field around the vehicles suggest that the differences in the velocity and volume of the entrained flow of the flap versus no-flap vehicles were due to pressure differences generated by the bell kinematics of the vehicles. The pressure field around the bell margin of the flap version had much lower pressure over a larger region than the no-flap version (Fig. 2B and inset). In fact, the pressure field extended from the bell tip to the inflexion point of the flexible margin (Fig. 2B) suggesting that the flow generated during bell contraction could be directly related and perhaps predicted by the dimensions of the propulsor inflexion.

**Figure 5:**
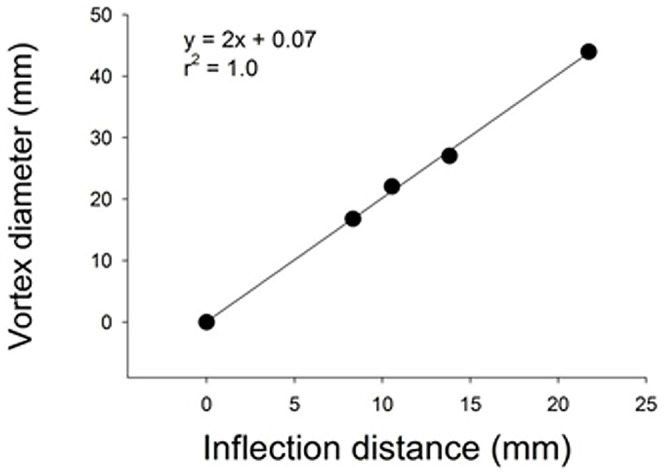
Flap length versus vortex diameter. Maximum diameter of the starting vortex versus the distance measured from the bell margin to the inflexion point (location where the flap joined the rigid actuator) for *Aurelia* vehicle with variable length flaps.

**Figure 6:**
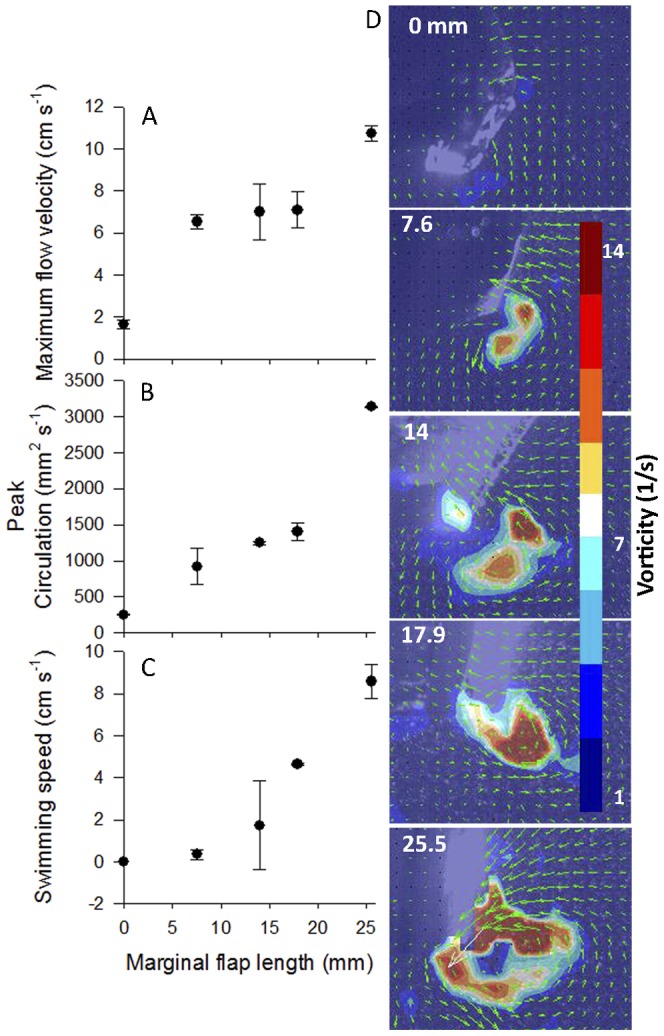
Fluid interactions and performance of vehicles with variable flap lengths. (A) Maximum fluid velocity and (B) peak circulation of the starting vortex for the *Aurelia* vehicle with variable length flaps. (C) Maximum swimming speed of the *Aurelia* vehicle with variable length margins. (D) Velocity vectors and vorticity contours of the starting vortex during bell contraction of the different versions of the vehicle.

To examine if inflexion dimensions relate directly to vortex generation we compared the flow generated by the robotic *Aurelia* vehicle with variable length flaps attached to the bell margin. This comparison demonstrated that the size of the vortex ring (with minimum vorticity of 4 s^−1^) was strongly related to the distance between the tip of the bell margin and the inflexion point (Fig. 5). In addition, we found that the size of the marginal flap was directly related to the maximum velocity of the flow entrained by the contracting bell margin (Fig. 6A) and, as a result, the overall circulation and swimming performance were also directly related to the marginal flap length (Fig. 6B and 6C).

**Figure 7:**
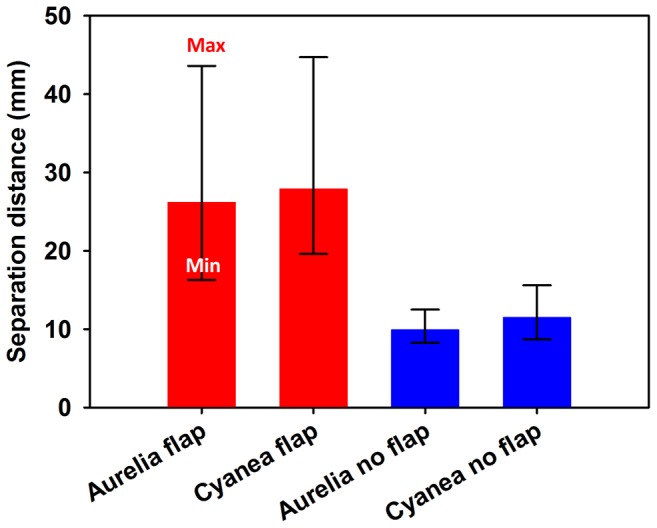
Vortex separation for flap and no-flap vehicles. Average distance between the starting and stopping vortex during bell expansion for the flap (red) and no-flap (blue) version of the vehicles. The error bars represent the range showing the closest and the furthest that the rings were in proximity throughout the recovery.

As mentioned earlier, swimming kinematics during bell expansion also contributed to the overall performance differences observed between the flap and no-flap vehicles. The deceleration during bell expansion was much lower for the flap versions than for the no-flap versions which contributed greatly to the differences observed in the overall forward progress of the robotic vehicles over time (Fig. 3). The deceleration of the no-flap robots was accompanied by a likewise cancellation of the circulation of the starting vortex (Fig. 3C). The cancellation of the circulation was caused by the collision of the starting vortex with the oppositely spinning stopping vortex generated during bell expansion (Fig. 7). In contrast, there was a much greater distance between the starting and stopping vortices for the flap versions of the vehicles (Fig. 7) which prevented vortex cancellation and recoil of the vehicle (Fig. 3C and 3D). In essence, the presence of the marginal flap appeared to more optimally control the spacing and interaction of the vortices around the pulsing robot vehicles.

**Figure 8:**
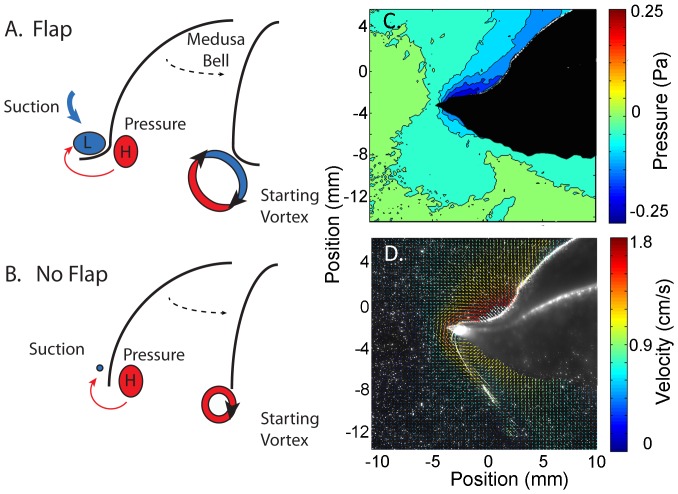
Proposed mechanism of thrust enhancement. Schematic of the proposed mechanistic basis of elevated circulation of vehicles with (A) flexible margins relative to those with (B) rigid margins. Kinematics of the flexible margin during bell contraction creates a large low pressure region between the bell margin and the inflexion point. This creates a suction that entrains high velocity flow in the region of the inflexion point. In contrast, higher pressure along the subumbrellar surface ejects fluid from under the bell. The difference in pressure between the two regions generates increased circulation by the extruded fluid that joins flow at the inflexion point to coalesce into a broader vortex at margins of bells with flexible flaps (A) relative to those without flexible flaps (B). (C) The pressure and (D) velocity vector fields around the bell margin of the scyphomedusa *Aurelia aurita* during bell contraction demonstrate the presence of a low pressure region extending from the bell margin to the inflexion point and maximum fluid velocities in the vicinity of the inflexion point, respectively.

**Figure 9:**
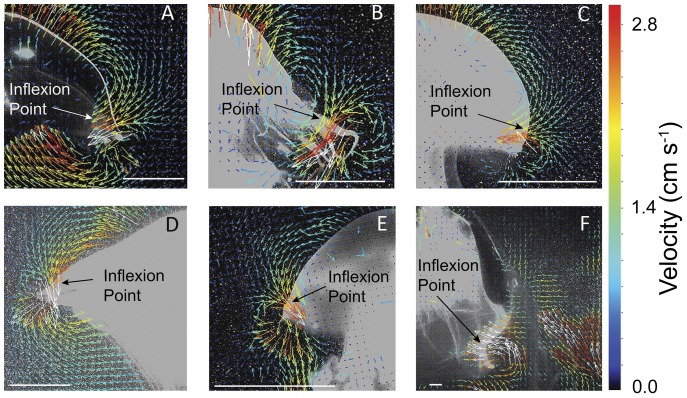
Flow around inflexion points of medusae. Flow during initial stages of bell contraction and vortex production at the bell margins of six medusan rowing swimmers. (A) *Aurelia aurita*, (B) *Chrysaora quinquecirrha*, (C) *Mastigias papua*, (D) *Cyanea capillata*, (E) *Phyllorhiza punctata*, (F) the velarium of a large (13 cm bell diameter) *Chironex fleckerii*. White scale bars represent a spatial reference of 1.0 cm. Note that, for all species, the highest velocity vectors are directed into the inflexion point at the bell margin as illustrated in Figure 8A.

The quantified pressure field and flow adjacent to the bell margin of the scyphomedusa *Aurelia aurita* resembled that of the flap vehicles and the fluid interactions observed around *A. aurita* were similar to the flap vehicles. Specifically, there was a low pressure region that extended from the tip of the bell margin to the inflexion point of the bell during contraction. As the bell continued to contract the inflexion point traveled toward the bell tip as a traveling wave. The low pressure region followed the inflexion point to the bell tip and became encircled by the starting vortex at the end of bell contraction. Consequently, the maximum flow contributing to the starting vortex was the entrained flow located at the inflexion point of the bell (Fig. 8C and 8D). In fact, velocity gradients found for the medusan vehicles resembled those of a variety of rowing-propelled medusae. DPIV analysis of flows around the bell margins of several species of scyphomedusae and one large cubomedusae (Fig. 9) demonstrated that maximum fluid velocities were located at the inflexion point of the bell margin during bell contraction. Video sequences demonstrated that the flows generated at the inflection point coalesced with fluid forced outwards from beneath the bell margin as the margin swept through bell contraction cycles. The similarity of fluid flows indicates that similar hydrodynamic processes occurred at both vehicle and living medusan bell margins.

## Discussion

The utilization of robotic vehicles to examine biological propulsion has provided insight into just how important flexible propulsors are for some swimming animals. These vehicles, in combination with the simple body plan of medusae, have also enabled us to visualize the structural differences in the fluid interactions of flexible and rigid propulsors. Visualizations are necessary to begin to understand the mechanistic basis of the enhancement in propulsion provided by flexible propulsors and begin applying these principles more generally. To summarize what can be taken away from these more or less preliminary observations of propulsion by robotic jellyfish, we found that flexibility significantly enhances the swimming performance of both the robotic vehicles examined (*Aurelia* and *Cyanea* versions). Furthermore, the enhanced performance was due to both thrust augmentation during bell contraction and to more optimal spacing between consecutive starting and stopping vortex rings during bell expansion. The strong resemblance of vorticity patterns generated by the robotic vehicles with those of living medusae supports the notion that the flexible bell margins of medusae function to enhance thrust similarly to the flaps of the robotic vehicles. These observations enable us to suggest some mechanisms by which thrust augmentation by flexible propulsors may guide future studies on this topic.

Much of the performance differences between the flap and no-flap vehicles can be attributed to the higher vortex circulation generated by flexible bell margins during bell contraction. Higher circulation is expected to augment performance because thrust production of a propulsor is proportional to the circulation generated at the propulsor margin [34], [36]. However, the mechanical basis of elevated vorticity surrounding the flexible flaps was unexpected because it was not consistent with previous computational models of vortex production by margin edges. Specifically, vortices have been envisioned as forming at the propulsor tip during its passage through the fluid [21], [25], [37] and their strength (circulation) directly reflects the velocity of the tip through the fluid. Models of vortex formation at the margins of foils, wings or fin surfaces [10], [25], [38], [39] reflect this pattern. However, the tips of our flexible vehicular margins did not travel at greater maximum or average velocities compared to rigid vehicle margins (Fig. 4). Yet circulation generated by flexible margins was substantially greater than those of the more rapidly moving, rigid margins. Additionally, greater circulation at flexible margins was not due to surface area differences for the vehicles due to the flaps, as demonstrated by circulation rates normalized by propulsor surface area (Fig. 3D).

Rather than the previously reported explanations, we suggest that increased circulation characterizing flexible bell margins was more likely due to unique hydrodynamic forces generated by bending at the inflexion point connecting the flexible margin to the more rigid actuating structure of the vehicle's bell. During bell contraction, a low pressure region extended along the exumbrellar surface from the bell margin to the inflexion point of the flap vehicles. This low pressure region was larger and lower in magnitude in the flap vehicles than the pressure along the bell margin of the no-flap vehicles. Consequently, entrained flow in this low pressure region at the inflexion point initiated vortex formation and merged with flow expelled from the inside the bell at the margin edge. The larger volume and velocity of this flow is directly related to the low pressure field and resulted in larger starting vortex formation at the bell margins, and hence, greater overall thrust production by vehicles with flexible margins. Although we have focused on empirical visualization and quantification of fluid flows surrounding vehicle margins, these results are consistent with computational fluid dynamic models describing pressure gradients surrounding swimming bells of oblate medusae. Swimming by oblate medusae generates pressure differences at the bell margin due to formation of a high pressure region within the medusa bell and a lower pressure region outside the bell margin at the region of greatest bell displacement [8].

These observations lead us to suggest that one way that flexible propulsors enhance performance of swimming jellyfish is by intensifying the low pressure region associated with the propulsor thereby increasing fluid flow and therefore overall thrust (Fig. 8). The relative positions of these high and low pressure regions and the resulting pressure gradients are likely determined by the geometry and kinematics of the inflexion point and may serve as a control mechanism for thrust production. In fact, it appears that the distance between the bell margin and the inflexion point may define the low pressure field and scale directly with the size of the vortex formed (Fig. 5). But more work is needed to confirm this detail of prediction. The low pressure field and flow at the inflexion point of the live jellyfish *Aurelia aurita* are consistent with the proposed mechanism by which flexible margins enhance swimming performance. Other mechanisms likely contribute to this simple pressure mechanism such as the angular motions associated with flexible flapping which may facilitate vortex formation [40]. However, the role of these pressure differences on either side of a flexible margin or tip have not been previously explored and may provide insight for future studies on the design of propulsor margins.

In addition to enhanced thrust generated during bell contraction, performance of the flap vehicles was also better during bell expansion. Vortex interactions during bell expansion appear to largely influence the overall swimming performance of the vehicles. The collision of the stopping vortex of the no-flap vehicle with the oppositely spinning starting vortex cancelled out the overall circulation in the wake and stopped the vehicle's forward progress. This observation suggests two things, first, that interactions between starting and stopping vortices play important roles in determining the medusan swimming performance. Consequently, adequate spacing of these vortices by propulsor kinematics appears to be essential for swimming. Second, the flexible propulsor spaced the vortices more optimally than the rigid propulsor. By increasing the spacing between the two vortices, the starting and stopping vortices did not cancel each other out and the circulation in the wake dissipated slowly due to viscous dissipation. Consequently, the flap vehicles continued their forward progression during bell expansion. Numerical simulations of other flexible propulsors support the notion that flexibility increases spacing between shed vortex rings by increasing the downstream advection of the vortices [12].

The absence of complicating fluid interactions, such as with additional fins or appendages, makes the simple morphologies of medusae useful for exploration of essential fluid interactions between a propulsor margin and surrounding fluids. Despite their simplicity, the fluid mechanical basis of enhanced vortex production by flexible medusan margins may have wider application to other biological propulsors. The suitability of the medusan bell margin model for comparison with other flexible propulsors is influenced by the extent to which a propulsor margin's bending creates an inflexion point near the margin. A variety of biological propulsors such as wings and fins fit these conditions and fundamental relationships between actuator force, margin dimensions, and flexural stiffness distributions may be important factors influencing the role that flexible margins play in propulsive force generation by propulsors of both natural and man-made design. A more full understanding of these interactions will allow us to appreciate the means by which animals convert the energetic liability that tip vortices present for fixed wing aircraft [41] into an asset for lift and thrust production by biological propulsors.
